# Improved detection of rice yellow mottle virus with a polyclonal antibody xMAP assay: A high-throughput alternative to ELISA

**DOI:** 10.1016/j.virusres.2026.199740

**Published:** 2026-05-05

**Authors:** A Vernet, G Thaurignac, N Poulicard, J Bouillin, A Pinel-Galzi, A Pinault, P K A Bamogo, M Peeters, A Ayouba, S Lacombe

**Affiliations:** a PHIM, Plant Health Institute of Montpellier, Univ. Montpellier, IRD, CIRAD, INRAE, Institute Agro, Montpellier, France; b TransVIHMI, Univ. Montpellier, IRD UMI 233, Inserm U1175, Montpellier, 34090, France

**Keywords:** RYMV, xMAP Luminex immunoassay, Polyclonal antibody, Diagnostic

## Abstract

Rice yellow mottle virus (RYMV) is one of the most destructive viral pathogens affecting rice production in sub-Saharan Africa, where its extensive genetic diversity and high evolutionary rate complicate reliable diagnosis. Current serological methods, notably double-antibody sandwich enzyme-linked immunosorbent assay (DAS-ELISA), are widely used for routine detection but may lack sensitivity when viral loads are low or samples are degraded. In this study, we developed and evaluated a high-throughput microsphere immunoassay (MIA) based on Luminex xMAP technology as an alternative serological tool for RYMV detection. A panel of three monoclonal antibodies (mABs) and one polyclonal antibody (pAB), all raised against a RYMV isolate from Madagascar, was assessed using greenhouse-propagated and field-collected rice samples representing all six known RYMV genetic groups. Assay performance was evaluated in terms of sensitivity, specificity, and accuracy using receiver operating characteristic (ROC) curve analysis, and results were compared with those obtained by DAS-ELISA. While mABs failed to retain their expected serotype specificity in the xMAP format, the polyclonal antibody enabled robust and strain-independent detection of RYMV. The pAB-based xMAP assay showed excellent diagnostic performance, with 100% specificity, sensitivity exceeding 97%, and an area under the ROC curve of 0.99. Importantly, the pAB-based xMAP assay demonstrated a 100- to 500-fold increase in sensitivity compared with DAS-ELISA, allowing reliable detection of RYMV in samples with low viral loads or compromised integrity. No cross-reactivity was observed with other co-circulating rice viruses, including maize streak virus and rice stripe necrosis virus. This study demonstrates that a polyclonal antibody-based xMAP immunoassay provides a highly sensitive, specific, and scalable alternative to conventional ELISA for RYMV diagnosis. This approach represents a promising tool for large-scale epidemiological surveys and improved management of rice viral diseases in Africa.

## Highlights

• A polyclonal antibody–based xMAP immunoassay enables highly sensitive detection of rice yellow mottle virus (RYMV).
• The assay detects all six RYMV genetic groups without cross-reactivity with other rice viruses.
• Sensitivity is 100–500 times higher than conventional DAS-ELISA, enabling detection in low-viral-load samples.

## 1 Introduction

Rice (*Oryza sativa* L.) is a staple food for over half of the world's population (Mohimed et al., 2022). Pathogens and pests, particularly rice viruses, pose one of the most serious threats to this cereal crop worldwide, causing significant yield loss. (Savary et al., 2019). To date, 19 species of rice virus have been identified that cause varying degrees of damage to rice production (Wang et al., 2022). Of these 19, two have long been reported in Africa: the rice yellow mottle virus (RYMV) (*Sobemovirus, Solemoviridae*) and the rice stripe necrosis virus (RSNV) (*Benyvirus, Benyviridae*) (Tucker et al. 2020; Bagayoko et al., 2021; Hébrard et al., 2021). More recently, the maize streak virus (MSV) (*Mastrevirus, Geminiviridae*) and the maize streak Reunion virus (MSRV; *Mastrevirus, Geminiviridae*) have been reported to infect rice in West Africa (Fouad et al., 2024; Bangratz et al. 2024). The symptoms of viral infections can be quite diverse, ranging from stunted growth to yellowing leaves, and can result in significant yield losses. Given the importance of rice as a food crop, accurate detection of rice viruses is crucial for disease management and control.

First recorded in Kenya in the 1960′s (Bakker et al., 1974), RYMV is one of the most damaging rice viruses and a major concern for Africa rice production. It is now prevalent in almost all sub-Saharan countries where rice is cultivated, causing major losses ranging from 20% to 100% in varieties adapted to lowland and irrigated ecosystems (Hébrard et al. 2021; Abrokwah et al., 2024). RYMV has non-enveloped, monopartite, icosahedral particles and a single-stranded RNA genome that is ca. 4450 nucleotides long. The virus is transmitted mechanically by insect vectors, particularly chrysomelids, and by contact between plants (Traoré et al. 2009). Infected plants exhibit characteristic yellow mottling on their leaves, as well as reduced tillering and stunting. Poor panicle exertion eventually leads to the premature death of the plant. The RYMV genome is organized in five overlapping open reading frames (ORFs) : ORF1, ORFx, ORF2a, ORF2b, and ORF4 (Ling et al. 2013; Hébrard et al. 2021). ORF1 and ORF4 sequences vary by strain, while ORF2a and ORF2b sequences are conserved (Fargette et al. 2004). ORF4, encoding the coat protein (CP), is mainly used for RYMV phylogenic analysis due to the high degree of diversity that allows strain discriminations (Pinel-Galzi et al. 2015). An in-depth analysis of RYMV diversity has revealed six main genetic groups, i.e. strains, which have been identified based on molecular and serological analysis. They are widely distributed across Africa, with a geographical distribution that follows distinct ecological zones. Strains S1, S2 and S3 comprise West African isolates, while S4, S5 and S6 contain isolates from East Africa (Pinel-Galzi et al., 2015; Ndikumana et al. 2025).

As RYMV is present in all areas of Sub-Saharan African countries where rice is grown and can cause devastating losses, accurate, high-throughput, timely detection of RYMV is critical for effective disease management and control. Various methods are employed for this purpose, ranging from visual inspection to advanced serological and molecular techniques. Visual diagnosis typically involves identifying characteristic RYMV symptoms. However, these methods lack specificity and require expertise in recognizing RYMV viral symptoms from those caused by other biotic or abiotic factors. The main method for serological detection of RYMV isolates is assessed using immunological tests, such as the double- or triple-antibody sandwich enzyme-linked immunosorbent assay (DAS-ELISA or TAS-ELISA) (N’Guessan et al., 2000; Fargette et al., 2002). RYMV polyclonal antiserum, prepared against an S4 isolate from Madagascar, is used as the primary antibody in DAS-ELISA for broad detection, but without serotype specificity. To determine RYMV strains, a TAS-ELISA is performed using a polyclonal antibody (pAB) as the primary antibody and specific monoclonal antibodies (mABs) as the secondary antibodies. mAB A reacts with strains S1, S4, S5 and S6, while mABs D and E react with all strains except S1 and S5, respectively (N'Guessan et al., 2000; Fargette et al., 2002). DAS-ELISA and TAS-ELISA are the main methods used for RYMV detection, as they are virus- and serotype-specific, sensitive, relatively straightforward and capable of processing multiple samples simultaneously. However, these methods can be limited in terms of sensitivity and specificity when dealing with low infection levels (beginning of infection, tolerant / resistant varieties) or degraded samples such as field samples poorly conserved or historical herbaria. A molecular method such as reverse transcriptase-polymerase chain reaction (RT-PCR), coupled with Sanger sequencing, offers a more sensitive insights into strain identification thanks to the amplification of the ORF4. qRT-PCR and next-generation sequencing (NGS) can provide more detailed insights into viral load and the identification of mixed infections, respectively, but they require expensive specialized equipment and/or bioinformatics expertise (Hébrard et al. 2021; Abrokwah et al. 2024; Bangratz et al. 2025).

Over the past 25 years, a new technology called xMAP (Multi-Analyte Profiling) has emerged. In addition to all the advantages of ELISA, this serological technique offers a reduced sample volume, increased sensitivity, greater flexibility, higher throughput, and lower cost, while maintaining a similar workflow (Fulton et al., 1997; Carson et al., 1999). xMAP technology is a microsphere array method that enables both simplex and multiplexed assays for protein applications. In the field of human health, the xMAP technology is used to detect specific antibodies, whereas in the field of plant health, it is used to detect specific antigens (for exemple Ayouba et al., 2017; Yu et al., 2018). Unlike ELISA, the capture antigen/antibody is covalently immobilized on fluorescently labelled magnetic beads. Following a reporting reaction, the Luminex analyzer excites the beads in order to classify each microsphere particle and to detect the fluorescent signal emitted by the reporter molecules captured during the assay. This technique therefore requires less capture antibody and smaller sample volumes, which is important when working with in-house antibodies and precious samples. Furthermore, non-specific antibody/antigen binding is significantly reduced (Peck et al., 2006; VanDerMeid et al., 2011; Liu et al., 2011).

In the domain of human health, the xMAP technology was initially used with limited sample types, such as cerebrospinal fluid and synovial fluid (de Jager et al., 2005). Since then, this analytical method has been developed and optimized to measure human antibodies, with varying degrees of success, particularly in large-scale, high-throughput surveys. xMAP technology has been used for the detection of HIV and Simian immunodeficiency virus antibodies (Ahuka-Mundeke et al., 2011), as well as for the sensitive and specific detection of antibodies to Zaire Ebola virus (Ayouba et al., 2017). It has also been applied to detect antibodies against infectious laryngotracheitis and bronchitis viruses (Wang et al., 2018). More recently, xMAP technology has been used to study the disease caused by the SARS-CoV-2 virus (Ayouba et al., 2020; Das and Dunbar, 2023). Luminex has licensed this method for use in biological assays to several kit developers operating in the clinical diagnostics, pharmaceutical and life science research markets. Taking into account factors such as cost-effectiveness, sensitivity, accuracy, the growing number of published studies, and the widespread use of commercial assays, the Luminex xMAP is a powerful and promising alternative to ELISA (Resolva et al., 2017).

In plant health diagnostics, one of the major challenges is the availability of specific antibodies. mABs development requires specialized facilities, significant financial investment, and time-consuming procedures, making them less accessible for routine use. As an alternative, pABs can be generated more quickly and at a lower cost, but their variability, specificity and potential cross-reactivity often limit their diagnostic performance in plant virus detection assays.

To date, only a few publications have reported the use of xMAP for the detection of plant pathogens. xMAP has been validated for the detection of the iris yellow spot virus (IYSV) in the *Nicotiana benthamiana* multiplication host. The sensitivity of this technique for detecting IYSV has been reported as 16-fold higher than ELISA (Yu et al., 2018). The xMAP technique has also been developed for detecting plum pox virus (PPV) in both the *N. benthamiana* multiplication host and the *Prunus domestica* natural host, with a sensitivity similar to ELISA (Croft et al., 2008). These two xMAP techniques rely on the use of mAbs to detect either IYSV or PPV. xMAP techniques have also been developed in a multiplex format for the detection of several viruses in potatoes, including potato virus X (PVX), potato virus Y (PVY) and potato leafroll virus (PLRV) (Bergervoet et al., 2008), as well as PVX, PVY and PLRV, plus potato virus S (PVS), potato virus A (PVA) and potato virus M (PVM) (Priegnitz et al., 2019). In both cases, the specificity of the multiplex xMAP techniques was similar to that of ELISA.

In this study, we present a high-sensitivity xMAP-based microsphere immunoassay for specific detection of RYMV in greenhouse- and naturally-infected samples. A set of three mABs and one pAB against RYMV were successfully tested. We compared the sensitivity and specificity of this method with those of the conventional DAS-ELISA, demonstrating that our xMAP approach provides superior performance. For the first time, we demonstrated that a polyclonal antibody can enable plant virus-specific detection across all serotypes with sensitivity 100–500 times greater than that of DAS-ELISA. This represents a significant opportunity to develop this method for the detection of plant viruses and for large-scale epidemiological surveys.

## 2 Material and methods

### 2.1 Plant material

The RYMV-positive and -negative plant samples used in this study were either obtained in a greenhouse after mechanical inoculation (Tables 1A and B) or collected directly from rice field after natural infection (Tables 2A and B).

**Table 1:** List of plant samples collected in greenhouse.

A				
Code	Name	Country of origin	Mutiplication date	Genetic group
S1*	BF2	Burkina Faso	2021	S1
S2*	CI63	Ivory Cost	2021	S2
S3*	CIa	Ivory Coast	2021	S3
S4*	Mg1	Madagascar	2021	S4
S5*	Tz3	Tanzania	2021	S5
S6*	Tz11	Tanzania	2021	S6
S1.1	CI3 (P11)	Ivoiry coast	2002	S1
S1.2	Ma10	Mali	2021	S1
S1.3	Ng106	Niger	2022	S1
S1.4	Tg247	Togo	2022	S1
S2.1	BF1	Burkina Faso	2022	S2
S2.2	CI11 (P37)	Ivoiry coast	2002	S2
S2.3	Ma4 (MC)	Mali	2003	S2
S2.4	Ma5 (M5)	Mali	2003	S2
S3.1	SL1	Sierra Leone	1998	S3
S3.2	SL103	Sierra Leone	2022	S3
S3.3	SL107	Sierra Leone	2022	S3
S3.4	SL111	Sierra Leone	2022	S3
S4.1	Mg16	Madagascar	2010	S4
S4.2	Tz5	Tanzania	2012	S4
S4.3	Tz8	Tanzania	2008	S4
S4.4	Tz201	Tanzania	2022	S4
S5.1	Tz2	Tanzania	2021	S5
S5.2	Tz9	Tanzania	2014	S5
S5.3	Tz10	Tanzania	2005	S5
S5.4	Tz211	Tanzania	2021	S5
S6.1	Tz18	Tanzania	2002	S6
S6.2	Tz21	Tanzania	2002	S6
S6.3	Tz202	Tanzania	2016	S6
S6.4	Tz209	Tanzania	2008	S6
**B**				
**Code**	**Name**	**species**	**Sampling date**	
Neg-1	Mg12	*O. glaberrima*	2024	
Neg-2	CSSL107	*O. sativa*	2024	
Neg-3	Caiapo	*O. sativa*	2024	
Neg-4	Kogoni A4	*O. sativa*	2024	
Neg-5	Kogoni A3	*O. sativa*	2024	
Neg-6	Kogoni B2	*O. sativa*	2024	
Neg-7	Kogoni Beta2	*O. sativa*	2024	
Neg-8	IR64	*O. sativa*	2024	
Neg-9	Kogoni	*O. sativa*	2024	
Neg-10	Mingui 63	*O. sativa*	2024	
Neg-11	Sanhuangzhan	*O. sativa*	2024	
Neg-12	Carolina Gold	*O. sativa*	2024	
Neg-13	IR64	*O. sativa*	2024	
Neg-14	Moroberekan	*O. sativa*	2024	
Neg-15	W1182	*O. latifolia*	2024	
Neg-16	Nipponbare	*O. sativa*	2024	
Neg-17	OG49	*O. glaberrima*	2024	
Neg-18	OB58S	*O. bartii*	2024	
Neg-19	Nerica L	*O sativa xO. glaberima*	2024	
Neg-20	2893	*O. sativa*	2024	
Neg-21	0001,712	*O. sativa*	2024	
Neg-22	0008,604	*O. sativa*	2024	
Neg-23	IK0714B	*O. sativa*	2024	

**A-** RYMV-positive samples were obtained via mechanical inoculation using an inoculum acquired from naturally infected leaves. Their code used in this study, their original name, their country of origin, the multiplication date and the RYMV genetic group of the initial inoculum are indicated. In this study, RYMV-positive samples are coded according to their genetic group.

* Indicates the samples that were used for the initial optimizations of the xMAP conditions.

**B-** RYMV-negative samples were collected in greenhouse in 2024. Their code used in this study, their original name and their species are indicated.

**Table 2:** List of plant samples collected in African rice field.

A				
Code	Name	Country of origin	Sampling date	Genetic group
BF_S1.1	BF114	Burkina Faso	2016	S1
BF_S1.2	BF179	Burkina Faso	2016	S1
BF_S1.3	BF216	Burkina Faso	2016	S1
BF_S1.4	BF141	Burkina Faso	2016	S1
BF_S1.5	BF182	Burkina Faso	2016	S1
BF_S1.6	BF133	Burkina Faso	2016	S1
BF_S1.7	BF13	Burkina Faso	2016	S1
BF_S1.8	BF41	Burkina Faso	2016	S1
BF_S1.9	BF124	Burkina Faso	2016	S1
Tz_S4	KPL150–4	Tanzania	2015	S4
Tz_S5	KPL126–41	Tanzania	2015	S5
Tz_S6.1	KPL290	Tanzania	2016	S6
Tz_S6.2	KPL346	Tanzania	2016	S6
Tz_S6.3	KPL76	Tanzania	2016	S6
Tz_S6.4	KPL158	Tanzania	2016	S6
Tz_S6.5	KPL332	Tanzania	2016	S6
SL_S3	SL218	Sierra Leone	2019	S3
SL_S2.1	SL219	Sierra Leone	2019	S2
SL_S2.2	SL230	Sierra Leone	2019	S2
SL_S2.S3	SL231	Sierra Leone	2019	S2/S3
**B**				
**Code**	**Name**	**Country of origin**	**Sampling date**	
BF_Neg.1	MP2824	Burkina Faso	2018	
BF_Neg.2	MP2825	Burkina Faso	2018	
BF_Neg.3	MP2829	Burkina Faso	2018	
BF_Neg.4	MP2831	Burkina Faso	2018	
BF_Neg.5	MP2830	Burkina Faso	2018	
BF_Neg.6	MP2841	Burkina Faso	2018	
BF_Neg.7	MP2845	Burkina Faso	2018	
BF_Neg.8	MP2842	Burkina Faso	2018	
BF_Neg.9	BZ14	Burkina Faso	2018	
BF_Neg.10	MP3017	Burkina Faso	2018	
BF_Neg.11	MP3021	Burkina Faso	2018	
BF_Neg.12	MP3029	Burkina Faso	2018	
BF_Neg.13	MP3030	Burkina Faso	2018	

**A-** RYMV-positive samples were collected in African fields based on viral leaf symptoms and verified by DAS-ELISA. Their code used in this study, their name, their country of origin, the sampling date and RYMV genetic group are indicated. In this study, RYMV-positive samples are coded according to their country of origin and their genetic group.

**B-** RYMV-negative samples were collected in Burkina Faso fields in 2018. Their code used in this study, their name and their country of origin are indicated.

The 30 RYMV-positive greenhouse samples were obtained after the susceptible *Oryza sativa indica* cultivar IR64 was mechanically inoculated with extract from leaf samples. The plant samples were collected two weeks after inoculation and verified by DAS-ELISA as soon as they were harvested, after which they were stored at −20 °C (Table 1A). Six samples (noted *) representing the six genetic groups were first used for the optimization of the xMAP conditions. Among the 24 other positive samples, the six genetic groups were represented by four samples per genotype. The original leaf samples used as inoculum were collected in eight West and East African countries (the Ivory Coast, Mali, Niger, Togo, Burkina Faso, Sierra Leone, Madagascar and Tanzania). Inoculum multiplication was performed between 1998 and 2022. The plant samples were collected two weeks after inoculation, tested by DAS-ELISA as soon as they were harvested, and subsequently stored at −20 °C as leaf sample (Table 1A).

The 23 RYMV-negative greenhouse samples were collected in 2024 and stored at −20 °C prior to use. They were cultivated in an S1 confinement greenhouse, which is a facility that is completely free of RYMV. The samples belonged to the *O. glaberrima, O. sativa, O. latifolia* or *O. bartii* species (Table 1B).

The 20 naturally infected RYMV samples were collected in rice fields between 2015 and 2019 from three African countries (Burkina Faso, Sierra Leone and Tanzania) (N. Poulicard pers comm). Sampling was performed based on visual observation of symptoms on the leaves, and the presence of RYMV was confirmed by DAS-ELISA. The symptomatic samples were first stored at room temperature during field survey and then at −80 °C in a notebook. The genetic group of each sample was determined by ORF4 sequencing. The RYMV-naturally infected samples selected here, included all six genetic groups (Table 2A).

The 13 RYMV-negative samples were collected from a rice field in Burkina Faso in 2018, as described by Billard et al. (2023). In each selected field, a 4 × 4 grid was marked out. The sixteen plants located at the grid nodes were visually inspected for symptoms and sampled in pools. Based on visual observation and DAS-ELISA, 13 samples were selected from the RYMV-negative pools. The samples were dried in silica gel and stored in an envelope at room temperature (Table 2B).

The three MSV samples collected in the greenhouse correspond to those described by Fouad et al. (2024). The four RSNV samples were collected in Ecuador field and verified by DAS-ELISA, RT-PCR and illumina sequencing (Paz Carasco et al. in prep). The co-infected MSV / RYMV sample was collected in a Burkina Faso field in the survey described in Fouad et al. (2024). It was verified by DAS-ELISA, RT-PCR and sanger sequencing.

### 2.2 Rice leaves treatment

Leaf samples consisting of 1 cm-long rice leaves were ground in liquid nitrogen and resuspended in 500 µl of phosphate-buffered saline (PBS) containing 0.05% Tween 20. After centrifugation at 7000 g for 10 min, the supernatant was recovered and stored at −20 °C prior to use.

### 2.3 Enzyme-linked immunosorbent assay detection of RYMV

Serological detection was performed using the double antibody sandwich enzyme-linked immunosorbent assay (DAS-ELISA), as described by N’Guessan et al. (2000), with a polyclonal antibody against an isolate from Madagascar. Absorbance at 405 nm was measured using a Spark multimode plate reader (Tecan). Following the methodology of Traoré et al. (2008), samples were considered positive if the optical density obtained was higher than the average for negative samples plus three times the standard deviation.

## 3 xMAP immunoassay detection of RYMV

### 3.1 Antibodies coupling to Luminex beads

The polyclonal antibody (pAB) and the three different monoclonal antibodies (mABs A, D and E) that we used in this study were prepared against an RYMV isolate from Madagascar (Fargette et al. 2002; N’Guessan et al. 2000). Antibodies (mABs and pAB) were covalently coupled on magnetic carboxyl-functionalized fluorescent polystyrene beads (Luminex Corp., Austin, TX) with the Bio-Plex Amine Coupling Kit (Bio-Rad Laboratories, Cat 171–406,001, Marnes-la-Coquette, France) according to the manufacturer’s instructions. For the optimization tests, two concentrations of antibodies (1 μg or 2 µg for 10^6^ beads) were compared.

### 3.2 Secondary antibody biotinylation

The pAB raised against RYMV was biotinylated with the Thermo Fisher EZ-Link Sulfo-NHS-LC-Biotin Reagent (Cat. No. PI-21,335) according to the standard procedure provided by the manufacturer. In brief, appropriate volume of Sulfo-NHS-LC-Biotin was added to the 1 mg of dialysis antiboby and incubated on ice for 2 h. The buffer of antibody-coupled biotin preparation was exchanged and the excess of biotin reagent was removed by using a desalting column. The success of the coupling reaction was evaluated by HABA (4′-hydroxyazobenzene-2-carboxylic acid) assay for measuring the level of biotin incorporation.

### 3.3 Microsphere immunoassay screening for RYMV

To develop our assay, we adapted a previously described xMAP-based immunoassay for the detection of SARS-CoV-2 antibodies (Ayouba et al., 2020). Protein-coupled microspheres were used at a concentration of 2,000 beads by well in assay buffer. Plant samples, pre-diluted 1:200, were incubated with the bead mixture in the dark under 400 rpm shaking for either 2 h at room temperature or 18 h at 4 °C. Viral particle binding was detected using the biotinylated polyclonal antibody, as previously described, diluted 1:10,000, followed by a 1 h incubation in the dark at 37 °C under 400 rpm shaking. Subsequently, streptavidin–R-phycoerythrin (SAPE) (Invitrogen) was added for test as the reporter reagent at two final concentrations of 1 and 4 µg/mL, with a 10 min incubation at room temperature. Data acquisition was performed on a BioPlex-200 system (Bio-Rad, Marnes-la-Coquette, France). Results are expressed as median fluorescence intensity (MFI) per 50 beads.

## 4 Calculation of cutoff, sensitivity, specificity, and accuracy

We used receiver operating characteristic (ROC) curve analysis to determine the cutoff values for each antibody used in the Luminex assay. The ROC curve analysis is a graphic representation of the relationship between both the sensitivity and specificity of a diagnostic test, on samples with known disease status. The cutoff is at the optimum where sensitivity and specificity curves intersect. The sensitivity was then defined as the ratio of number of samples found to be positive with the assay to the number of true positives, and the specificity was defined as the ratio of number of samples found to be negative with the assay to the number of true negatives. The accuracy of the assay was defined as the number of correct assessments with the assay divided by the number of all assessments. The accuracy can also be deduced from ROC curve analysis by the value of the area under the curve (AUC). The ROC curve analysis was performed using GraphPad Prism version 10.0.0 for Windows, GraphPad Software, Boston, Massachusetts USA.

## 5 Comparison between DAS-ELISA and xMAP in detecting RYMV

The sensitivity of DAS-ELISA and xMAP in detecting RYMV was evaluated using the same set of RYMV-positive greenhouse samples. The initial extracts were diluted in PBS buffer to generate dilution series of factors 10 from 10^−3^ to 10⁻⁸ for both the DAS-ELISA and xMAP assays. The samples were tested using DAS-ELISA and xMAP with pAB, as described above.

## 6 Results

### 6.1 Optimization of antibodies and SAPE substrate condition

**Fig. 1:**
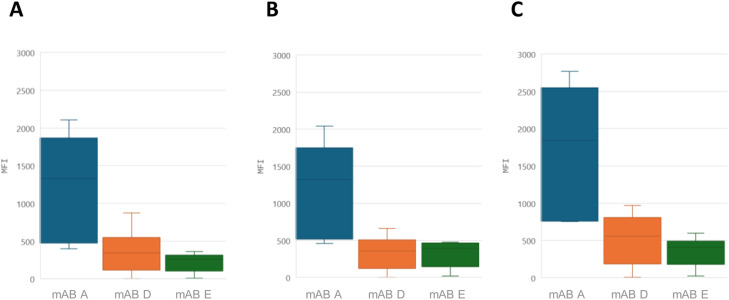
Setting the amount of antibodies and SAPE substrate concentration for xMAPassays using the monoclonal antibodies mAB A, mAB D and mAB E. A- xMAP assays were performed with the antibodies titrated at 1 µg per 1 × 10⁶ beads and with SAPE substrate concentration set to 1 µg/ml. B- xMAP assays were performed with the antibodies titrated at 2 µg per 1 × 10⁶ beads and with SAPE substrate concentration set to 1 µg/ml. C- xMAP assays were performed with the antibodies titrated at 1 µg per 1 × 10⁶ beads and with SAPE substrate concentration set to 4 µg/ml. xMAP assays were performed on greenhouse-propagated rice leaf samples (six RYMV-positive; see Table 1) tested at a dilution of 1:100. The box plots show median fluorescence intensity (MFI) distributions for 50 beads per antibody.

We used the three mABs A, D and E as capture antibody to set up the xMAP assay. Six greenhouse-propagated RYMV-positive rice samples, covering all six serotypes, were used at a dilution of 1/200 (v/v) (Table 1). Increasing the incubation time of the samples/beads from 2 h to overnight improved the MFI values for all six samples without significant concomitant background increase (data not shown). This overnight incubation time was used for subsequent experiments. We then, optimized the conditions for the mABs using either 1 µg or 2 µg per 10⁶ beads (Fig. 1A and 1B). Regardless of the amount of coupled mABs, no changes in MFI signal intensity was observed. Thus, we chose 1 µg per 10⁶ cells as the optimal cost-effective condition. Then, the effect on reaction efficiency of SAPE substrate concentrations of either 1 or 4 µg/ml was evaluated (Fig. 1A and 1C). An improved MFI signal intensity was observed for all three mABs at a SAPE concentration of 4 µg/ml. Therefore, this concentration was chosen for all subsequent experiments.

### 6.2 Specificity of monoclonal antibodies in the xMAP assay

**Fig. 2:**
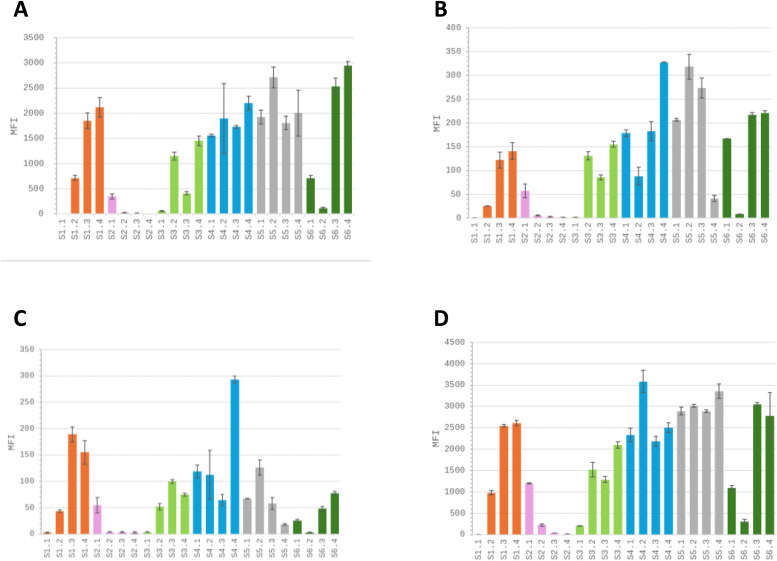
A comparison of the efficiency of xMAP assays using monoclonal antibodies (mABs A, D and E)and apolyclonal antibody (pAB) to detect RYMV-positive samples. A- xMAP assays were performed on 24 RYMV-positive samples, representing all six serotypes (four different samples per serotype), using the mAB A monoclonal antibody as the capture antibody. B- Same experiments were performed using mAB D monoclonal antibody as the capture antibody. C- Same experiments were performed using mAB E monoclonal antibody as the capture antibody. d- Same experiments were performed using the pAB as the captureantibody. MFI values represent the mean of two independent experiments. Error bars indicate standard deviations. Each graph has its own scale for MFI values. The orange bars represent the S1 samples. The pink bars represent the S2 samples. The pale green bars represent the S3 samples. The blue bars represent the S4 samples. The grey bars represent S5 samples. The dark green bars representthe S6 samples.

The mABs A, D and E were previously characterized for their serotype-specific response when used in a triple-antibody-sandwich ELISA (TAS-ELISA) (Pinel et al., 2002). mAB A recognised all serotypes except S2 and S3; mAB D recognised all serotypes except S1; and mAB E recognised all serotypes except S5. To evaluate whether these serotype specificities were maintained in xMAP experiments, the assays were performed on 24 greenhouse-propagated RYMV-positive rice samples, representing the six serotypes (Table 1A). MFI signals were stronger when mAB A was used as the capture antibody, compared with mAB s D and E, regardless of the serotype, with signal intensities reaching 2900 (Fig. 2A, 2B and 2C).

The weakest MFI signals were obtained with the four S2 samples, regardless of the mAB used, whereas this was only expected with mAB A according to TAS-ELISA specificities. Serotype-specific responses were not observed with any of the mAB s used. Furthermore, MFI signal profiles were similar for all serotype samples, regardless of the mAB used in the xMAP assays with weak MFI signals for S1.1, S2.2, S2.3, S2.4, S3.1 and S6.2 samples. This suggests that differences in MFI signal intensity would be due to variable virus concentrations rather than mAB serotype specificity.

To verify this hypothesis, the polyclonal antibody (pAB), which is classically used in DAS-ELISA for serotype-independent RYMV diagnostics, was used as the capture antibody in an xMAP assay on the same set of 24 RYMV-positive rice samples (Fig. 2D). For each sample, the MFI signals obtained with this pAB were higher than those obtained with mAB s, ranging from 9 to over 3500. The pattern of MFI signal intensity was similar to those obtained with mAB s A, D and E, supporting the idea that the differences in MFI signals were due to variable virus concentration between samples. These data demonstrate that mABs lose their serotype specificity when used as a capture antibody in an xMAP assay, and that the pAB is more efficient than mAB s in detecting RYMV-positive samples, regardless of serotype, in an xMAP assay. Furthermore, the signal threshold must be determined in order to accurately distinguish between positive and negative samples, particularly those with low MFI values.

### 6.3 Cut-off calculation

To determine the cut-off point to distinguish between positive and negative samples for mABs A, D and E and pAB used in xMAP assays, we tested 36 RYMV-negative and 44 RYMV-positive samples, representing all six strains, at a dilution of 1:200 under the conditions described above (Tables 1 and 2). The samples were obtained from greenhouse-propagated plants and field samples collected across three African countries. Receiver operating characteristic (ROC) curve analysis was used to determine the cut-off values for the four antibodies, identifying the optimal cut-off point that maximizes the sensitivity and specificity of the assay (Fig. Supp.).

The calculated cut-off values for MFI/50 beads for mABs A, D and E, and for pAB, were 1.25, 4.75, 3.75 and 6.5, respectively, with respective areas under the curve (AUC) of 0.9808, 0.7858, 0.8474 and 0.9878, respectively. Specificity was 97.87% for the three mABs and 100% for the pAB. Sensitivity varied from 54.17% to 97.92%, with the highest level being achieved with the pAB. These values reflect excellent discrimination between RYMV-positive and -negative samples, particularly for mAB A and the pAB, which is indicative of near-perfect diagnostic accuracy (Supp data).

### 6.4 Discrimination between RYMV-negative and RYMV-positive samples using xMAP assay

**Fig. 3:**
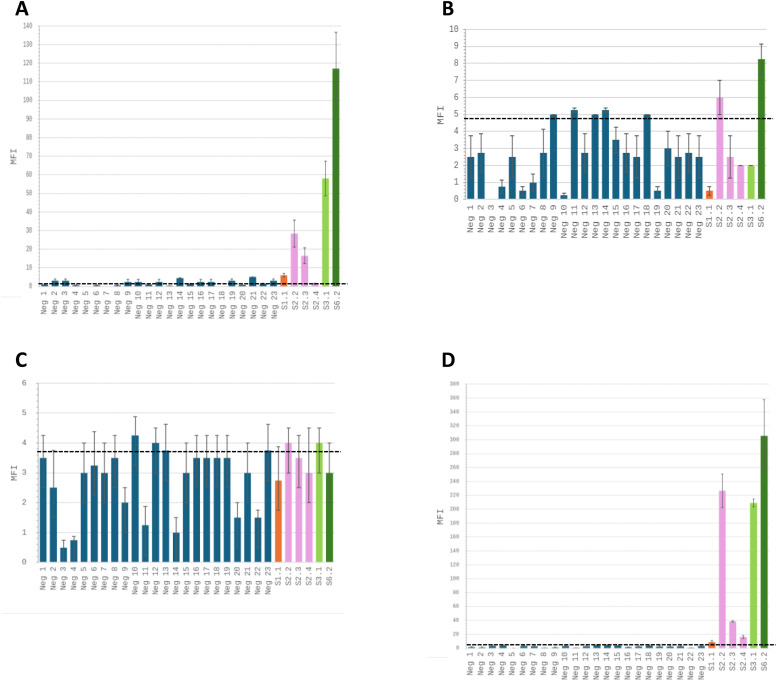
A comparison of the efficiency of xMAP assays using monoclonal antibodies (mABs A, D and E) and the pAB in distinguishing between RYMV-negative and low concentration RYMV-positive samples. A- xMAP assays were performed on 23 RYMV-negative samples plus the six RYMV-positive samples displaying the weakest MFI values, using the mAB A monoclonal antibody as the capture antibody. B- Same experiments were performed using mAB D monoclonal antibody as the capture antibody. C- Same experiments were performed using mAB E monoclonal antibody as the capture antibody. d- Same experiments were performed using the pAB as the captureantibody. MFI values represent the mean of two independent experiments. Error bars indicate standard deviations. The horizontal dotted black lines indicated the cut-off values. Each graph has its own scale for MFI values. The dark blue bars represents the negative samples. The orange bar represent the S1 sample. The pink bars represent the S2 samples. The pale green bar represent the S3 sample. The dark green bar representthe S6 sample.

To evaluate which antibody is the more efficient in discriminating RYMV-negative and RYMV–positive samples using the xMAP technique, xMAPs assays were performed on 23 RYMV-negative samples obtained in greenhouse (Table 1B) and on RYMV-positives samples displaying the weakest MFI signal in previous experiments. For each antibody, the samples were classified as positive or negative based on their MFI and the antibody cut-off value (Fig. 3). For mAB A, two negative samples (Neg 14 and Neg 21) were misclassified as positive, while one positive sample (S2.4) was misclassified as negative (see Fig. 3A). Other negative samples displaying MFI values close to the cut-off value (between 2.5 and 3) could be considered uncertain. All negative samples for mABs D and E displayed MFI values under or very closed to the cut-off value (Fig. 3B and C). However, four out of six positive samples and six out of six positive samples were misclassified as negative using mAB D and mAB E, respectively. Using the pAB, all negative and positive samples were correctly classified. These data suggest that the pAB is more effective at discriminating between RYMV-negative and RYMV-positive samples under our working conditions than mABs.

### 6.5 Sensitivity of xMAP assay using the polyclonal antibody in detecting RYMV

The pAB is classically used in the DAS-ELISA test as a routine RYMV diagnostic tool. To compare the sensitivity of the xMAP and DAS-ELISA assays in detecting RYMV, the same set of the 24 greenhouse RYMV-positive samples was analysed using both techniques. Unexpectedly, 11 out of the 24 samples tested negative using the newly performed DAS-ELISA, despite being classified as positive using the initial DAS-ELISA test performed directly after sampling. All 24 positive samples displayed an MFI above the cut-off value using the xMAP assay (Figs. 2D and 3D). RT-PCRs were performed on the 11 samples that were negative for DAS-ELISA and positive for xMAP. They confirmed the positive status for RYMV infection (data not shown). Nine of the 11 DAS-ELISA misclassified samples were collected before 2004, whereas all of the DAS-ELISA positive samples were collected after 2005. This suggests that the duration of sample storage and/or the number of freeze-thaw cycles may affect sample quality, reducing the sensitivity of the DAS-ELISA to RYMV detection without affecting the sensitivity of the xMAP assay.

For samples classified as positive using both the xMAP assay and the newly performed DAS-ELISA, the same set of serial dilutions was performed to identify their detection threshold using both techniques (Table 3). Depending on the sample, the sensitivity of the xMAP assay was 100–500 times higher than that of the DAS-ELISA assay. All these data demonstrate that the xMAP technique using the RYMV pAB is a more efficient and sensitive tool for detecting RYMV in greenhouse rice samples than the classical DAS-ELISA assay.

**Table 3:** Comparison of the dilution thresholds for DAS-ELISA and xMAP for the detection of RYMV.

Code	ELISA threshold	xMAP threshold	ELISA/xMAP factor
S1.2	5 × 10^−3^	10^−5^	500
S1.3	5 × 10^−3^	10^−5^	500
S1.4	10^−3^	10^−5^	100
S2.1	10^−3^	10^−5^	100
S3.2	5 × 10^−3^	10^−5^	500
S3.3	10^−3^	10^−5^	100
S3.4	5 × 10^−3^	10^−5^	500
S4.3	5 × 10^−3^	10^−5^	500
S4.4	10^−3^	10^−5^	100
S5.1	5 × 10^−3^	10^−5^	500
S5.2	10^−3^	10^−5^	100
S5.3	5 × 10^−3^	10^−5^	500
S6.4	5 × 10^−3^	10^−5^	500

### 6.6 Efficiency of the xMAP assay on field rice samples

To evaluate the efficiency of the xMAP assay set up with the pAB in detecting RYMV in field rice samples, assays were performed on sets of 20 and 13 samples collected in African rice fields and classified as positive or negative, respectively, based on RYMV symptoms and DAS-ELISA assays (Table 2A and B). This set of RYMV-positive samples included all six serotypes.

**Fig. 4:**
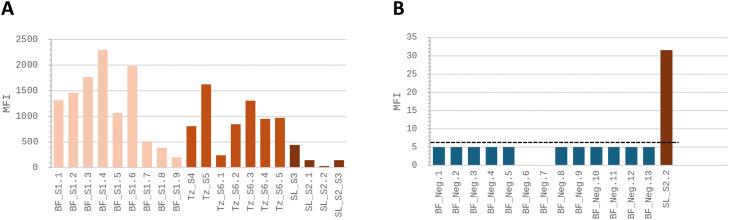
Detection of RYMV in field rice samples collected in West Africa using xMAP with the pAB. A- xMAP assays were performed on 20 RYMV-positive field samples. Nine were collected in Burkina Faso (BF), seven in Tanzania (Tz) and four in Sierra Leone (SL). The corresponding serotype is indicated. B- xMAP assays were performed on 13 RYMV negative field samples plus SL_S2.2 positive sample displaying the weakest MFI value among RYMV-positive samples. The horizontal dotted black lines indicated the cut-off values. Each graph has its own scale for MFI values. The pale orange bars represent the positive samples from Burkina Faso. The medium orange bars represent the positive samples from Tanzania. The dark orange bars represent the positive samples from Sierra Leone. The dark blue bars represent the negative sample from Burkina Faso.

The MFI values obtained for the set of RYMV-positive samples were all above the cut-off value of 6.5. These values ranged from 32 to 2300 (see Fig. 4A), which is consistent with the samples' RYMV-positive status. The MFI values obtained for the RYMV-negative samples were all below the cut-off value, ranging from 0 to 5 (see Fig. 4B), which confirms their negative status, as determined by previous symptom observation and DAS-ELISA. Altogether, these results demonstrate the efficiency of the xMAP assays we set up with the RYMV pAB in discriminating between RYMV-positive and RYMV-negative rice field samples.

### 6.7 Specificity of xMAP assay

To evaluate the efficiency of the xMAP assay set up with the RYMV pAB for the specific detection of RYMV, assays were performed on samples infected with either MSV or RSNV. These two viruses were chosen because they were detected in the same African regions as RYMV (Tucker et al. 2020; Bagayoko et al. 2021; Fouad et al. 2024). One sample infected with both RYMV and MSV was also included in the assay. All MSV- and RSNV-positive samples displayed MFI values below the cut-off value of 6.5, whereas the RYMV/MSV co-infected sample was detected as positive with an MFI value of 432 (Table 4). These results demonstrate that the xMAP assay set up with the RYMV pAB does not cross-react with other co-circulating viruses as MSV and RSNV.

**Table 4:** Evaluation of the xMAP assay's specificity in detecting RYMV using the RYMV polyclonal antibody.

Code	Virus	Host	Origin	MFI mean
MSV.1	MSV-A	*O. sativa*	greenhouse	3.5
MSV.2	MSV-G	*O. sativa*	greenhouse	3
MSV.3	MSV-A	*Z. mays*	greenhouse	2.5
RSNV.1	RSNV	*O. sativa*	Ecuador field	3.5
RSNV.2	RSNV	*O. sativa*	Ecuador field	3.5
RSNV.3	RSNV	*O. sativa*	Ecuador field	5
RSNV.4	RSNV	*O. sativa*	Ecuador field	3.5
co-infection	MSV / RYMV	*O. sativa*	Burkina Faso field	432

The assay was performed on samples infected with MSV, RSNV, or both RYMV/MSV. The obtained MFI values are indicated alongside the host and origin of the samples.

## 7 Discussion

Accurate virus diagnosis is crucial for epidemiological survey and effective disease management and control. Here, we report the development of serological tool based on xMAP Luminex technology for the first time for the detection of the major virus infecting rice in Africa, namely RYMV.

The efficiency of three monoclonal and one polyclonal antibody was evaluated using a set of positive and negative greenhouse samples representing the six serotypes. Using monoclonal mAB A and polyclonal antibodies produced the best discrimination between positive and negative samples, with only 3 and 0 out of 47 samples being misclassified, respectively. However, none of the mABs produced the expected serotype-specific responses observed in TAS-ELISA tests. These observations could be explained by differences in antigen capture and detection between the xMAP and ELISA formats. In the xMAP assay, mABs are coupled at high density to the microspheres, allowing antigen capture through multiple simultaneous binding events. Although individual antibody–antigen interactions may exhibit low affinity, their cumulative effect results in high avidity, stabilizing the complex during the assay. This multivalent binding can therefore retain antigens from different serotypes through weak but additive interactions. In addition, the fluorescence-based detection used in the xMAP system is more sensitive than the colorimetric detection used in ELISA, enabling the measurement of low-intensity signals that would remain undetected in ELISA. Together, these factors contribute to the apparent loss of serotype specificity observed with mABs in the xMAP format.

The xMAP test that we set up using the pAB enabled the accurate detection of all positive samples, whether they were mechanically (greenhouse) or naturally (field) infected, whereas the ELISA test performed on the same samples misclassified some of the positive samples. The dilution threshold was up to 500 times lower for xMAP than for ELISA. Furthermore, we demonstrated the specificity of the xMAP test, as MFI values obtained for samples infected with either the co-circulating MSV or RSNV viruses were below the cut-off. Together, these data demonstrate that our xMAP test using the pAB is sensitive and specific for diagnosing RYMV, even in positive samples with a low viral load due to conservation issues or a low level of infection, or samples infected with other viruses.

Polyclonal antibodies have already been used in xMAP tests to detect viruses in plants (Bergervoet et al., 2008; Priegnitz et al., 2019). Bergervoet et al. (2008) demonstrated on potato samples that xMAP detection of PVY or PLRV was 10 times more sensitive than the ELISA test, whereas the ELISA test was more sensitive than xMAP in detecting PVX. Priegnitz et al. (2019) reported similar sensitivities when detecting six potato viruses, including PVY, PLRV and PVR, using either the xMAP or DAS-ELISA methods. The sensitivity of the xMAP test we developed using the pAB is 100- to 500-fold higher than that of classical DAS-ELISA. Despite having a similar duration (1.5 days) and cost (0.55 cents per sample) to the ELISA test, the xMAP test we developed is much more valuable for diagnosing RYMV due to its sensitivity. Moreover, the xMAP cost per sample can only decrease by multiplexing, which is one of the strength of this approach.

RYMV has been detected in rice cultivation areas across Sub-Saharan African countries. Its well-described genetic diversity revealed a spatial distribution with strains specific to West and East Africa (Pinel-Galzi et al., 2015). It has been demonstrated that RYMV evolves at a rate comparable to that of animal viruses, enabling it to adapt rapidly (Fargette et al., 2008). RYMV can efficiently overcome genetic resistance in rice, as demonstrated by the emergence of resistance-breaking isolates under controlled conditions. (Fargette et al., 2002; Pinel-Galzi et al., 2016; Bonnamy et al., 2022). Moreover, the intensification of rice cultivation and increased material exchange in Africa over the past decade has led to changes in the distribution areas of RYMV strains. This results in areas of overlap, such as Ghana and Burkina Faso, where S1 strains originating from West and Central Africa coexist with S2 strains (Omiat et al., 2023; Billard et al., 2023), at both the field and individual plant levels (Billard et al., 2023; Bangratz et al., 2025). Taken together, these findings suggest that RYMV strains display high and dynamic levels of diversity across African rice cultivation areas. Consequently, the xMAP test that we developed using the pAB is particularly well-suited to the detection of RYMV, as it enables the sensitive, virus-specific and generic detection of RYMV infection, regardless of the strain.

This general detection would be greatly completed by the use of other tools that can distinguish between RYMV strains. The xMAP technology used in a nucleic acid format, with virus-strain-specific oligonucleotide primers coupled to the xMAP beads has been used for the specific detection of plant virus strains. xMAP assays specific to the tomato yellow leaf curl virus and the cucumber mosaic virus were developed on tomatoes and *Nicotiana benthamiana*, respectively (van Brunschot et al., 2014; Bald-Blume et al., 2017a, 2017b). As the genetic diversity of RYMV is well characterised (Pinel-Galzi et al., 2015), strain-specific nucleic acid xMAP tools could be developed to complement the generic xMAP tool developed here. This would provide an integrated approach for effective RYMV disease management and control.

In addition to being highly sensitive and specific, xMAP technology offers the significant advantage of multiplexing, enabling the simultaneous detection of multiple analytes within a single sample. This approach allows the parallel analysis of several dozen distinct targets, thereby improving diagnostic efficiency, sample utilization, and analytical robustness. The xMAP multiplex assay has been widely developed for human health purposes (Ayouba et al., 2016, 2020; Wang et al., 2018), as well as for the simultaneous detection of viruses in plants (Bergervoet et al., 2008; Priegnitz et al., 2019). Three other rice viruses have been reported in Africa in the same area as RYMV (Fouad et al., 2025; Bagayoko et al., 2021). Here, we demonstrate that the xMAP RYMV diagnostic tool, which was developed using a pAB, does not cross-react with the two most prevalent of them, namely MSV or RSNV. ELISA tests to detect MSV and RSNV using specific pABs have been previously reported (Peterschmitt et al., 1990; Tucker et al., 2020; Maurino et al., 2018). The efficiency of these pABs should be evaluated using xMAP technology to detect MSV and RSNV in rice samples, first in simplex and then in multiplex to allow the simultaneous diagnosis of the major viruses known to circulate in African rice fields.

## 8 Conclusion

We have developed an original, specific and sensitive diagnostic tool against one of the most serious viruses affecting rice production in Africa: the rice yellow mosaic virus (RYMV). This tool is based on xMAP technology and uses a polyclonal antibody. It is much more sensitive than the classical DAS-ELISA method used for routine testing, making it a more accurate detection tool for degraded or low-infected samples. Moreover, it does not cross-react with other rice viruses circulating in Africa. Thanks to the advantages of xMAP multiplexing, this work paves the way for using this technology as a new serological method to detect simultaneously various rice viruses known to circulate in Africa.

**Declaration of generative AI and AI-assisted technologies in the manuscript preparation process**

During the preparation of this work, the authors used DeepL Write to edit the manuscript in English. After using this tool, the authors reviewed and edited the content as needed and take full responsibility for the content of the published article.

## CRediT authorship contribution statement

**A Vernet:** Writing – review & editing, Writing – original draft, Methodology, Data curation. **G Thaurignac:** Writing – review & editing, Methodology, Formal analysis, Data curation, Conceptualization. **N Poulicard:** Writing – review & editing, Resources, Methodology, Conceptualization. **J Bouillin:** Writing – review & editing, Methodology, Data curation. **A Pinel-Galzi:** Writing – review & editing, Methodology, Data curation, Conceptualization. **A Pinault:** Writing – review & editing, Methodology, Data curation. **P K A Bamogo:** Writing – review & editing, Funding acquisition, Conceptualization. **M Peeters:** Writing – review & editing, Funding acquisition, Conceptualization. **A Ayouba:** Writing – review & editing, Methodology, Funding acquisition, Conceptualization. **S Lacombe:** Writing – review & editing, Writing – original draft, Project administration, Methodology, Funding acquisition, Conceptualization.

## Declaration of competing interest

The authors declare that they have no known competing financial interests or personal relationships that could have appeared to influence the work reported in this paper.

## Data Availability

Data will be made available on request.

## Appendix Supplementary materials

Image, application 1
