# Supplementary material for: Improved detection of rice yellow mottle virus with a polyclonal antibody xMAP assay: A high-throughput alternative to ELISA
Source: Virus Res. 2026 May 5;368:199740. doi: 10.1016/j.virusres.2026.199740 (PMC13195790; doi:10.1016/j.virusres.2026.199740)
Supplement: Supplementary file 1 [file mmc1.pdf]

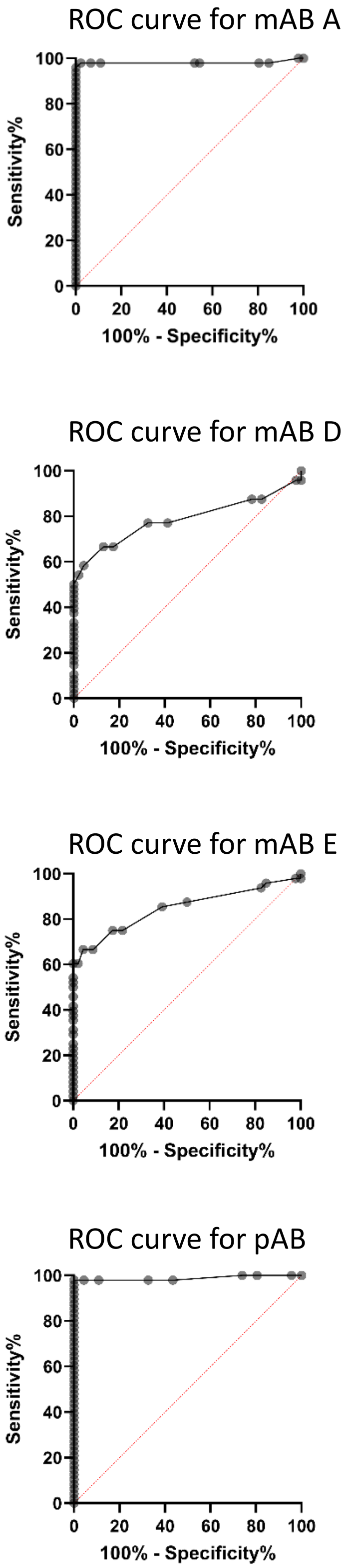

Supplementary data: Receiver operation characteristic (ROC) curves evaluating the discriminatory ability of the xMAP assays to differentiate between RYMV-negative and RYMV-positive samples for each antibody.
